# Distinct roles of TRAF6 and TAK1 in the regulation of adipocyte survival, thermogenesis program, and high-fat diet-induced obesity

**DOI:** 10.18632/oncotarget.22575

**Published:** 2017-11-03

**Authors:** Yann S. Gallot, Joseph D. McMillan, Guangyan Xiong, Kyle R. Bohnert, Alex R. Straughn, Bradford G. Hill, Ashok Kumar

**Affiliations:** ^1^ Department of Anatomical Sciences and Neurobiology, University of Louisville School of Medicine, Louisville, Kentucky 40202, USA; ^2^ Diabetes and Obesity Center, University of Louisville School of Medicine, Louisville, Kentucky 40202, USA

**Keywords:** obesity, adipocytes, signaling, glucose metabolism, brown adipose tissue

## Abstract

Chronic low-grade inflammation, adipocyte hypertrophy, and glucose intolerance are common features of obesity and a risk factor for cancer. Tumor necrosis factor (TNF) receptor-associated factor 6 (TRAF6) is an adaptor protein that also possesses a non-conventional E3 ubiquitin ligase activity. In response to receptor-mediated events, TRAF6 activates transforming growth factor-activated kinase 1 (TAK1), which leads to activation of the MAPK and nuclear factor-kappa B (NF-κB) signaling pathways. However, the roles of TRAF6 and TAK1 in the regulation of adipocyte function remain less understood. Here, we demonstrate that adipocyte-specific deletion of TAK1, but not TRAF6, in mice reduces the survival of adipocytes and abundance of white adipose tissue (WAT). Adipocyte-specific ablation of TAK1, but not TRAF6, increases the expression for markers of beige/brown fat in WAT. Deletion of TAK1 in WAT increases phosphorylation of AMPK, abundance of PGC-1α, non-canonical NF-κB signaling, markers of M2 macrophages, and diminishes phosphorylation of JNK and canonical NF-κB signaling. Levels of TRAF6 and enzymatic activity of TAK1 are increased in WAT of mice fed with high-fat diet (HFD). Our results demonstrate that ablation of TAK1 drastically reduces HFD-induced obesity and improves energy expenditure and glucose metabolism. In contrast, adipocyte-specific ablation of TRAF6 has a minimal effect on HFD-induced obesity. Collectively, our results suggest that even though TRAF6 is an upstream activator of TAK1 in many signaling cascades, inactivation of TAK1, but not TRAF6, regulates adipocyte survival, energy expenditure, and HFD-induced obesity in mice.

## INTRODUCTION

Obesity is a health care crisis of global pandemic status that is caused by a chronic imbalance between energy intake and expenditure [1]. Obesity and associated metabolic syndromes are the leading cause for type 2 diabetes (T2D), hypertension, cardiovascular disease, non-alcoholic fatty liver disease, osteoarthritis, and various types of cancer [1, 2]. Adipose tissue is broadly classified as either white adipose tissue (WAT) or brown adipose tissue (BAT). WAT is the major energy storage depot characterized by large lipid droplets and is a prominent endocrine organ, producing hormones that regulate feeding and satiety [3]. By contrast, BAT is an energy dissipation depot characterized by numerous mitochondria and high levels of expression of uncoupling protein-1 (UCP1). Recently, a third type of adipose tissue, called beige or brite fat, has been observed interspersed within WAT [4]. Like BAT, beige adipocytes express high levels of UCP1 and have a high energy expenditure capacity [3, 4]. Beige adipocytes can be induced by cold exposure, stimulated by certain chemicals and hormones, and by various genetic manipulations [3, 5–7]. Indeed, it is now evident that increasing the abundance of beige adipocytes is an approach that could prevent and treat obesity and T2D [8]. Nevertheless, the signaling mechanisms that regulate the acquisition of beige properties by WAT remain poorly understood.

Adipose tissue inflammation is a major characteristic of diet-induced obesity and is a critical link between obesity and the development of insulin resistance [9–11]. Pathological expansion of adipose tissue is caused by excessive adipocyte hypertrophy, which is associated with cell-autonomous hypoxia and activation of cellular stress pathways, such as: c-Jun N-terminal kinase (JNK), nuclear factor-kappa B (NF-κB), and endoplasmic reticulum stress-induced unfolded protein response pathways [12–14]. Activation of these pathways results in the increased expression and release of inflammatory cytokines and chemokines such as interleukin (IL)-6, tumor necrosis factor (TNF) α, monocyte chemoattractant protein 1 (MCP-1), and IL-12 [14, 15]. Locally secreted cytokines and chemokines attract proinflammatory macrophages into the adipose tissue, where they form crown-like structures around large or dying adipocytes. Excessive accumulation of adipose tissue macrophages further activates the inflammatory program in neighboring adipocytes, thereby exacerbating systemic inflammation and insulin resistance [12, 16].

TRAF6 is an upstream signaling protein that links a wide variety of cell surface receptors including Toll-like receptors (TLRs) and TNF superfamily receptors to intracellular signaling proteins to provoke inflammatory response [17, 18]. The N-terminal RING domain of TRAF6 is required for its ability to transmit signal. This domain functions as an E3 ubiquitin ligase which, together with the ubiquitin conjugating enzyme complex Ubc13/Uev1A, catalyzes the synthesis of a unique polyubiquitin chain linked through the lysine-63 (K63) residue in ubiquitin [19, 20]. TRAF6-generated K63-Ub oligomers activate TAK1 (also called MAP3K7) *in vitro* [18]. TAK1 is a central kinase that mediates the activation of several proinflammatory as well as pro-survival signaling pathways in a variety of cell types [21]. TAK1 makes a complex with TAK1-binding protein 1 (TAB1) and either TAB2 or TAB3 [22–25]. The specific interaction of the K63-Ub chains with the C-terminal domains of TAB2 and TAB3 induces a conformational change that leads to the activation of TAK1 [26]. TAK1 phosphorylates the MAPK kinases, MKK4 and MKK3/6, which leads to the activation of JNK1/2 and p38 MAPK, respectively, and eventually activates the proinflammatory transcription factor, Activator Protein 1 (AP-1) [18]. TAK1 also phosphorylates inhibitor of kappa B (IκB) kinase β (IKKβ) leading to the activation of NF-κB transcription factor through its canonical pathway [27]. AP-1 and NF-κB either alone or in combination induce the expression of a large number of cytokines, chemokines, and other inflammatory molecules [28].

In addition to their pivotal role in the inflammatory immune response, TRAF6 and TAK1 play critical roles in development and homeostasis of multiple tissues mainly through regulating cell survival, proliferation, and differentiation [18, 29]. However, the role of TRAF6 and TAK1 in regulation of adipocyte development and function remains less understood. A recent study demonstrated that tamoxifen-inducible adipocyte-specific deletion of TAK1 in mice reduces obesity, potentially through diminishing the survival of adipocytes and induction of browning in white adipose tissue [30]. Moreover, bone morphogenetic protein-mediated TAK1 signaling is required for the terminal differentiation of adipocytes [31]. While the *in vivo* role of TRAF6 in adipocytes has not been investigated, a recent study has demonstrated that miR146 suppresses the inflammatory response in human adipocytes by downregulating the levels of TRAF6 and interleukin-1 receptor-associated kinase 1 [32]. Although TLRs and many other receptor-mediated signaling events activate TAK1 through the upstream activation of TRAF6 [18], it remains unknown whether these two molecules have similar or distinct functions in the regulation of adipose tissue development and high-fat diet (HFD)-induced obesity.

Using genetic mouse models, we investigated the effects of adipocyte-specific deletion of TRAF6 or TAK1 in the development of adipose tissue and HFD-induced obesity. Our results demonstrate that deletion of TAK1, but not TRAF6, reduces the amount of WAT and increases the markers of BAT and beige fat in visceral adipose tissues. Deletion of TAK1 increased the phosphorylation of AMPK, increased levels of PGC-1α, and activated the non-canonical NF-κB pathway. Deletion of TAK1 drastically reduced HFD-induced obesity by diminishing adipocyte number and size. Ablation of TRAF6 had a comparatively lessened impact on HFD-induced obesity and it did not affect the survival of adipocytes. Our study suggests that TRAF6 and TAK1 differentially regulate adipocyte survival, white to beige adipocyte transition, and HFD-induced obesity.

## RESULTS

### Generation and characterization of adipocyte-specific TRAF6-knockout (KO) and TAK1-KO mice

**Figure 1:**
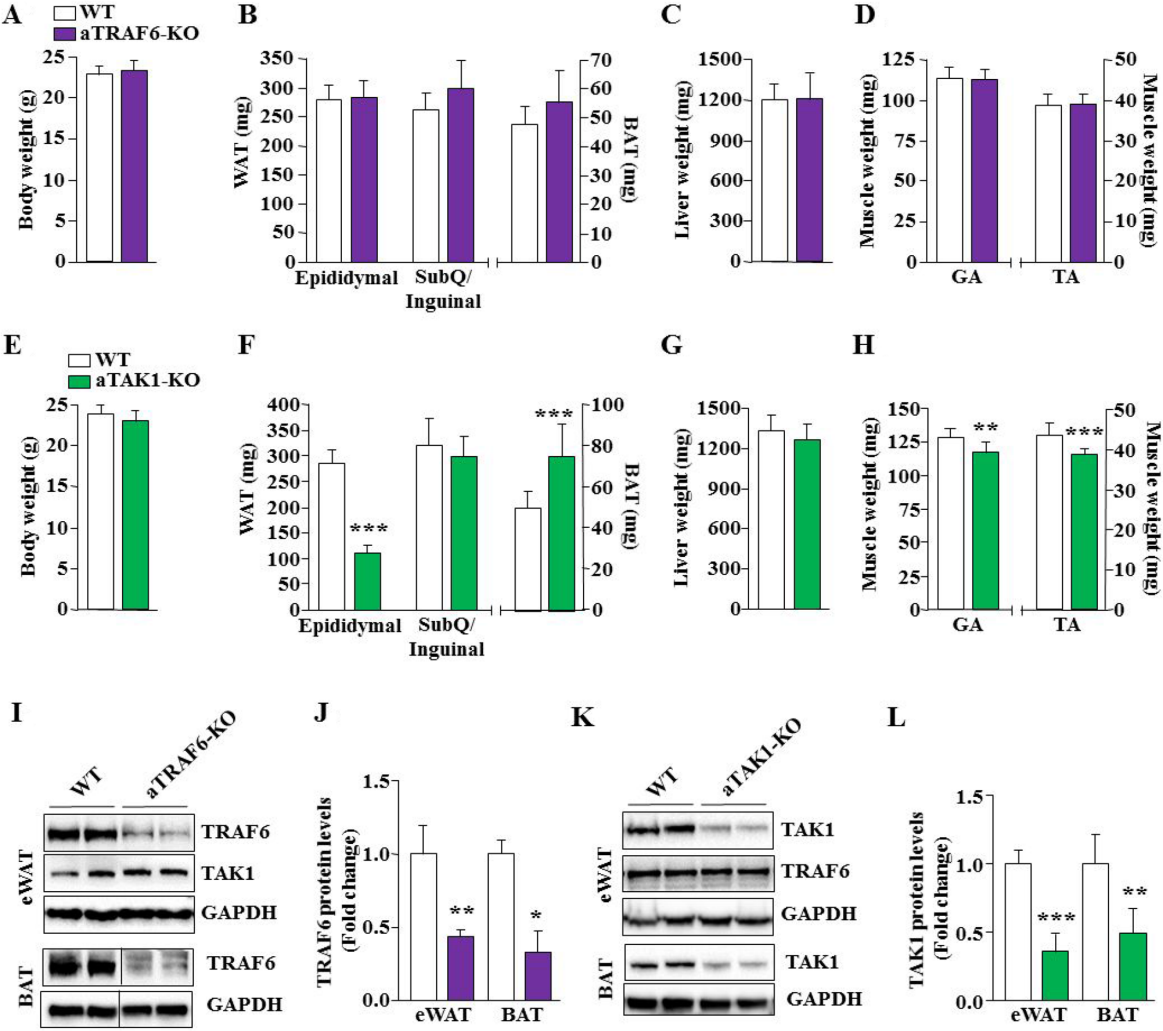
Effect of adipocyte-specific deletion of TRAF6 and TAK1 on white and brown fat mass (**A**) Overall body weight of 9-week old WT and aTRAF6-KO mice. (**B**) Quantitative analysis of wet weight of epididymal and subcutaneous (SubQ)/inguinal white adipose tissue (WAT), and interscapular brown adipose tissue (BAT) from WT and aTRAF6-KO mice. Wet weight of (**C**) liver, (**D**) gastrocnemius (GA), and tibialis anterior (TA) muscles of 9-week old littermate WT and aTRAF6-KO mice. *N* = 9 for WT mice and *n* = 5 for aTRAF6-KO mice. (**E**) Overall body weight of 9-week old WT and aTAK1-KO mice. (**F**) Quantitative analysis of wet weight of epididymal and SubQ/inguinal WAT and BAT from WT and aTAK1-KO mice. Wet weight of (**G**) liver, (**H**) GA and TA muscle of 9-week old WT and aTAK1-KO mice. *N* = 10 for WT mice and *n* = 9 for aTAK1-KO mice. (**I**) White and brown adipose tissues were isolated from WT and aTRAF6-KO mice and the levels of TRAF6, TAK1, and unrelated protein GAPDH were measured by performing Western blot. Vertical black line indicates that intervening lanes were spliced out. (**J**) Densitometry analysis of TRAF6 levels in eWAT and BAT of WT and aTRAF6-KO mice (*n* = 3–4 in each group). (**K**) Levels of TAK1, TRAF6, and unrelated protein GAPDH in eWAT from WT and aTAK1-KO mice. (**L**) Densitometry analysis of TAK1 levels in WAT and BAT of WT and aTAK1-KO mice (*n* = 4 in each group). ^*^p < 0.05, ^**^p < 0.01, and ^***^p < 0.001 from corresponding littermate WT mice by unpaired *t*-test.

To understand the role of TRAF6 and TAK1 in the development of adipose tissue, we generated mice in which TRAF6 or TAK1 was explicitly ablated in adipocytes. Specifically, floxed TRAF6 [33] or floxed TAK1 [34] mice were crossed with adiponectin (Adipoq)-Cre mice to generate adipocyte-specific TRAF6 knockout (henceforth aTRAF6-KO) and adipocyte-specific TAK1 knockout (henceforth aTAK1-KO) mice. Littermate floxed TRAF6 or floxed TAK1 mice served as wild-type (WT) control mice for aTRAF6-KO or aTAK1-KO mice, respectively. The mice were euthanized at the age of 9 weeks. Epididymal and subcutaneous (SubQ)/inguinal WAT and interscapular brown adipose tissue (BAT) as well as other organs were isolated and weighed. There was no significant difference in body weight (Figure 1A) or wet weight of epididymal and SubQ/inguinal WAT or BAT (Figure 1B), liver (Figure 1C) and hind limb muscle mass (Figure 1D) between WT and aTRAF6-KO mice. There was also no significant difference in overall body weight between WT and aTAK1-KO mice (Figure 1E). However, the wet weight of epididymal WAT (eWAT), but not SubQ/inguinal WAT, was significantly reduced whereas wet weight of interscapular BAT was significantly increased in aTAK1-KO mice compared with their littermate control mice (Figure 1F). While adipocyte-specific deletion of TAK1 did not affect wet weight of liver (Figure 1G), we found a small but significant decrease in wet weight of gastrocnemius (GA) and tibialis anterior (TA) muscles of aTAK1-KO mice compared with their WT littermates (Figure 1H). The exact mechanisms responsible for reduced muscle mass in aTAK1-KO remain unknown, however, a few possibilities can be discussed. It is plausible that the deletion of TAK1 reduces the amounts of adipocyte-derived myokines resulting in reduced muscle growth. Indeed, it is well-known that adipose tissue is a highly active metabolic and endocrine organ that regulates the homeostasis and metabolic function of multiple organs including: liver, bone and skeletal muscle [5, 35]. Alternatively, it is possible that the deletion of TAK1 leads to the increased production of inflammatory cytokines which causes muscle wasting. Our immunoblotting analysis confirmed that the levels of TRAF6 were significantly reduced in both WAT and BAT of aTRAF6-KO mice compared with corresponding WT mice (Figure 1I, 1J). Similarly, levels of TAK1 were significantly reduced in both WAT and BAT of aTAK1-KO mice compared with their controls (Figure 1K, 1L). Furthermore, there was no significant change in the levels of TAK1 in WAT of aTRAF6-KO and TRAF6 in WAT of aTAK1-KO mice compared to WT mice (Figure 1I, 1K).

### Targeted ablation of TAK1, but not TRAF6, reduces the number of adipocytes in eWAT

**Figure 2:**
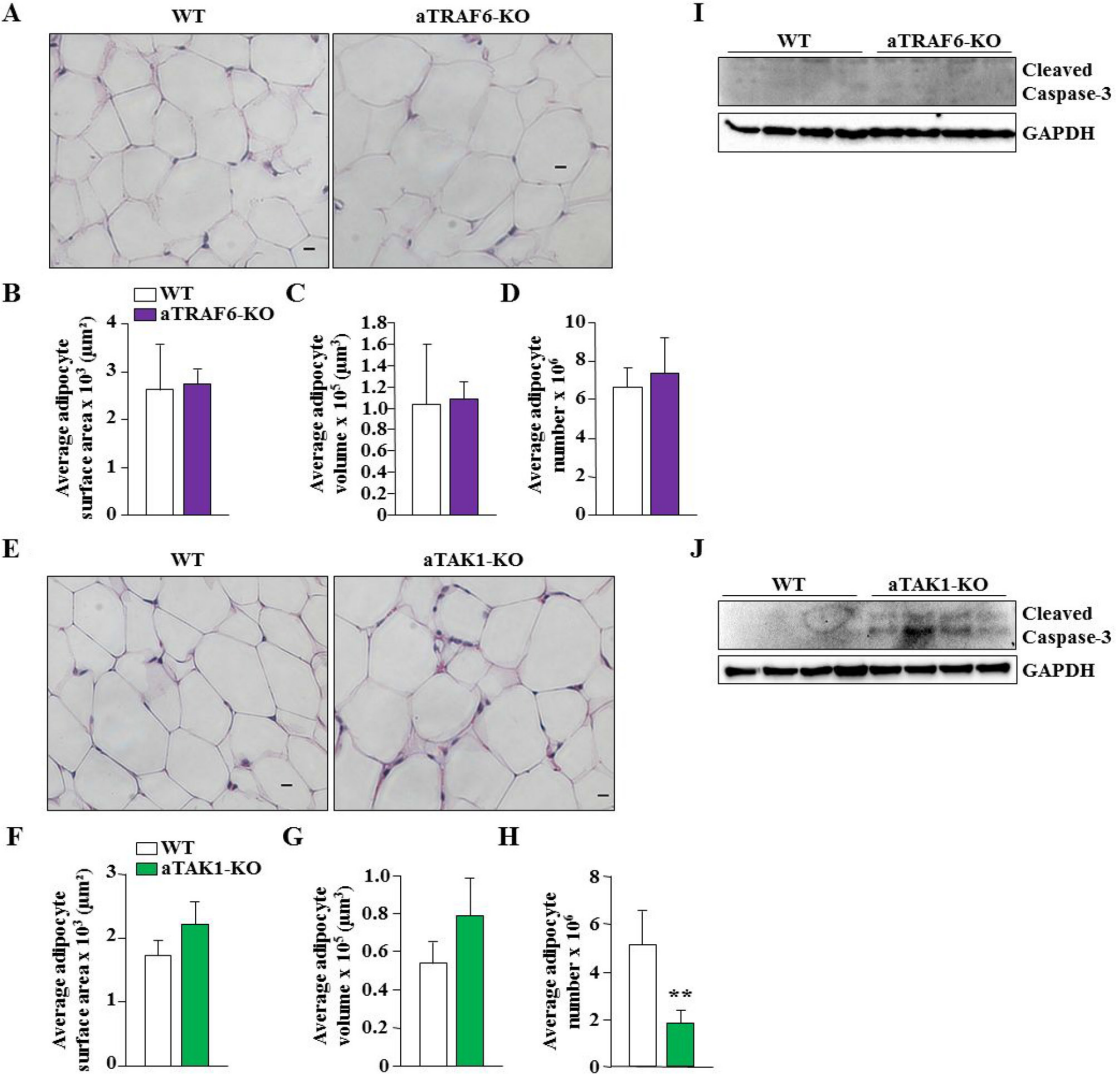
Ablation of TAK1 but not TRAF6 reduces adipocyte survival in eWAT of mice (**A**) Representative H&E-stained images of eWAT from 9-week old WT and aTRAF6-KO mice. Scale bars: 20 μm. (**B**) Average adipocyte surface area, (**C**) average adipocyte volume, and (**D**) average adipocyte number in eWAT of WT and aTRAF6-KO mice (*n* = 3 in each group). (**E**) Representative H&E-stained images of eWAT from 9-week old WT and aTAK1-KO mice. Scale bars: 20 μm. (**F**) Average adipocyte surface area, (**G**) average adipocyte volume, and (**H**) average adipocyte number in eWAT of WT and aTAK1-KO mice (*n* = 3 in each group). ^**^p < 0.01 from WT mice by unpaired *t*-test. (**I**) eWAT was isolated from WT and aTRAF6-KO mice and the levels of cleaved caspase-3 and unrelated protein GAPDH were measured by performing Western blot (*n* = 4 in each group). (**J**) Levels of cleaved caspase-3 and unrelated protein GAPDH in eWAT from WT and aTAK1-KO mice (*n* = 4 in each group).

To understand whether TRAF6 or TAK1 regulates adipocyte number or their size, we made paraffin sections of eWAT isolated from aTRAF6-KO and aTAK1-KO mice and their corresponding WT mice and performed hematoxylin and eosin (H&E) staining. There was no significant difference in the average adipocyte surface area, volume, or number in eWAT of WT and aTRAF6-KO mice (Figure 2A–2D). Furthermore, there was also no significant difference in average adipocyte surface area or volume in eWAT of WT and aTAK1-KO mice (Figure 2E–2G). However, the number of adipocytes was found to be significantly reduced in aTAK1-KO mice compared to WT mice (Figure 2H). TRAF6 and TAK1 can affect cell survival through regulating the activation of various pro-survival pathways [18, 29, 36]. Although the role of TRAF6 in the survival of adipocytes has not been documented, a recent study has shown that tamoxifen-inducible inactivation of TAK1 promotes apoptosis in adipocytes [30]. We measured the levels of cleaved caspase-3, a marker of apoptosis, in eWAT of aTRAF6-KO and aTAK1-KO mice and their littermate WT mice. Cleaved capsase-3 was not detected in eWAT of WT or aTRAF6-KO mice (Figure 2I). By contrast, cleaved caspase-3 was present in eWAT of aTAK1-KO mice (Figure 2J). These results suggest that the ablation of TAK1, but not TRAF6, reduces the amount of eWAT, potentially through diminishing the survival of adipocytes.

### Deletion of TAK1, but not TRAF6, exacerbates expression of inflammatory molecules in eWAT of mice

**Figure 3:**
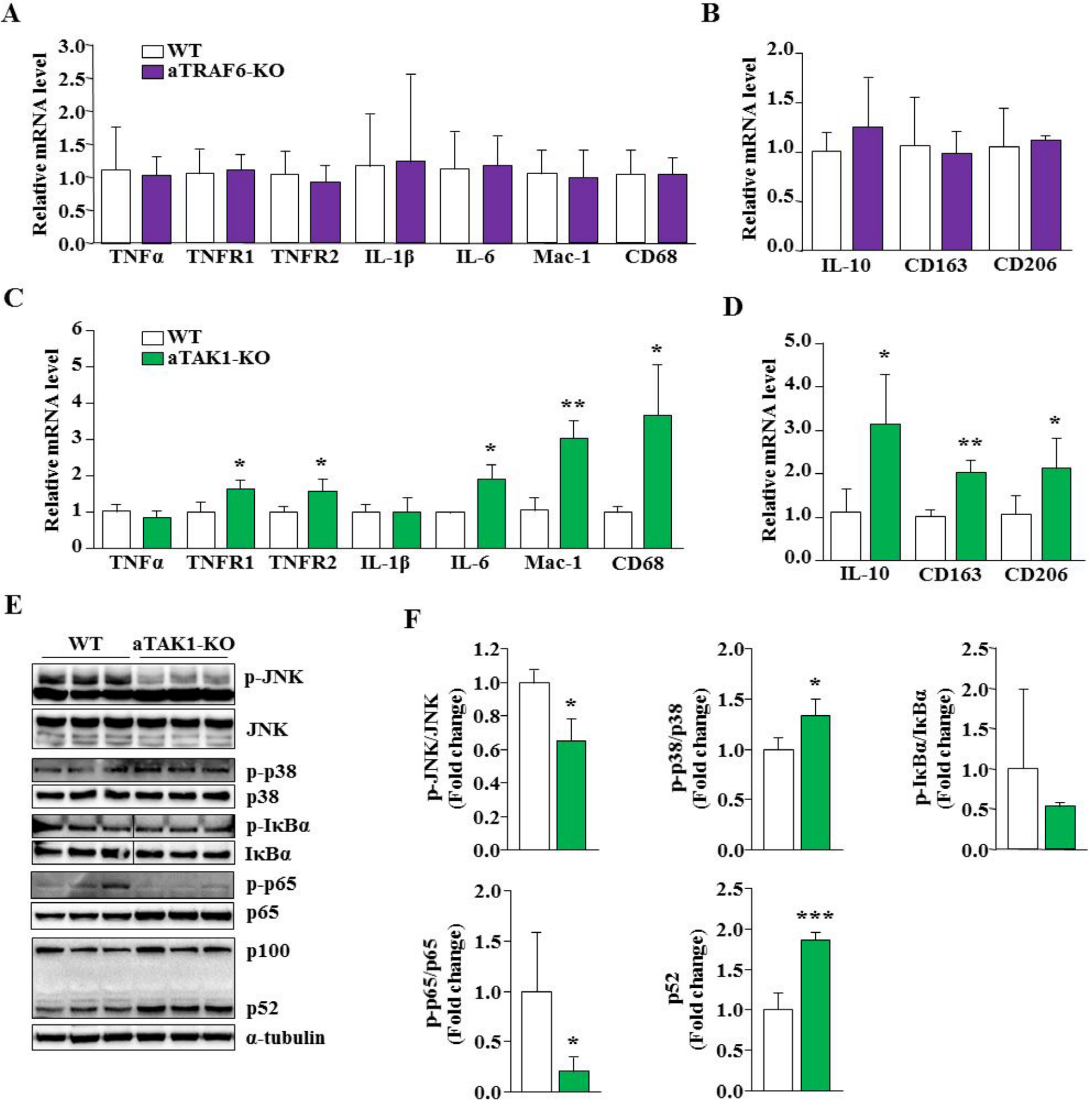
Effect of targeted ablation of TRAF6 and TAK1 on the expression of inflammatory molecules and signaling pathways (**A**) eWAT was isolated from 9-week old WT and aTRAF6-KO mice and relative mRNA levels of various inflammatory molecules and M1 macrophages were determined by performing QRT-PCR (*n* = 3-5 per group). (**B**) Relative mRNA levels of IL-10 and M2 specific markers in eWAT from WT and aTRAF6-KO mice (*n* = 3–5 per group). (**C**) eWAT was isolated from 9-week old WT and aTAK1-KO mice and mRNA levels of various inflammatory molecules and M1 macrophages were determined by QRT-PCR assay (*n* = 3–4 per group). (**D**) Relative mRNA levels of IL-10 and M2 macrophage specific markers in eWAT from WT and aTAK1-KO mice (*n* = 3–4 per group). Protein lysates were prepared from 9-week old eWAT of WT and aTAK1-KO mice, and blotted for specific signaling proteins. (**E**) Immunoblots, and (**F**) densitometry quantification of bands are presented here (*n* = 3 in each group). Vertical black line indicates that intervening lanes were spliced out. ^*^p < 0.05, ^**^p < 0.01, and ^***^p < 0.001 from corresponding WT mice by unpaired *t*-test.

By performing QRT-PCR, we next investigated whether deletion of TRAF6 or TAK1 affects the expression of inflammatory molecules in adipose tissue in naïve conditions. There was not a significant difference in the transcript levels of TNFα, TNF receptor (TNFR) 1, TNFR2, IL-1β, and IL-6 between the eWAT of WT and aTRAF6-KO mice. Moreover, the mRNA levels of macrophage markers, Mac-1 and CD68; M2 specific markers, CD163 and CD206; and IL-10 remain comparable between control and aTRAF6-KO mice (Figure 3A, 3B). In contrast, we found that mRNA levels of TNFR1, TNFR2, IL-6, Mac-1, and CD68 were significantly increased in eWAT of aTAK1-KO mice compared to their littermate controls (Figure 3C). There was not a significant difference between mRNA of TNFα and IL-1β between control and aTAK1-KO mice (Figure 3C). Intriguingly, we also found that mRNA levels of IL-10 and cell surface markers of M2 macrophages were significantly elevated in eWAT of aTAK1-KO mice compared to WT mice (Figure 3D). These results suggest that the inactivation of TAK1, but not TRAF6, perturbs the expression of various inflammatory molecules in eWAT of mice in naïve conditions. Moreover, these results suggest that deletion of TAK1 improves the levels of M2 macrophages in eWAT.

Since TAK1 but not TRAF6 reduces the amount of eWAT and disrupts the expression of inflammatory molecules, we focused our analysis on aTAK1-KO mice to determine whether the deletion of TAK1 affects the phosphorylation of specific signaling proteins that are implicated in inflammatory response and cell survival. Results showed that phosphorylation of c-Jun N-terminal kinase (JNK) was significantly reduced, whereas the phosphorylation of p38 MAPK was significantly increased in eWAT of aTAK1-KO mice compared to controls (Figure 3E, 3F). Moreover, we found that the phosphorylation of IkBα or p65 proteins, markers of activation for the canonical NF-κB pathway [28], were reduced in eWAT of aTAK1-KO mice compared to littermate WT mice (Figure 3E, 3F). Interestingly, the proteolytic processing of p100 protein into p52 protein, a marker for the activation of non-canonical NF-κB signaling [28, 37], was significantly increased in eWAT of aTAK1-KO mice compared to WT mice (Figure 3E, 3F). Collectively, these results suggest that while the ablation of TAK1 inhibits JNK and canonical NF-κB signaling, it leads to the increased activation of p38 MAPK and non-canonical NF-κB signaling in eWAT of mice in naïve conditions.

### Deletion of TAK1 causes white to beige fat transition

**Figure 4:**
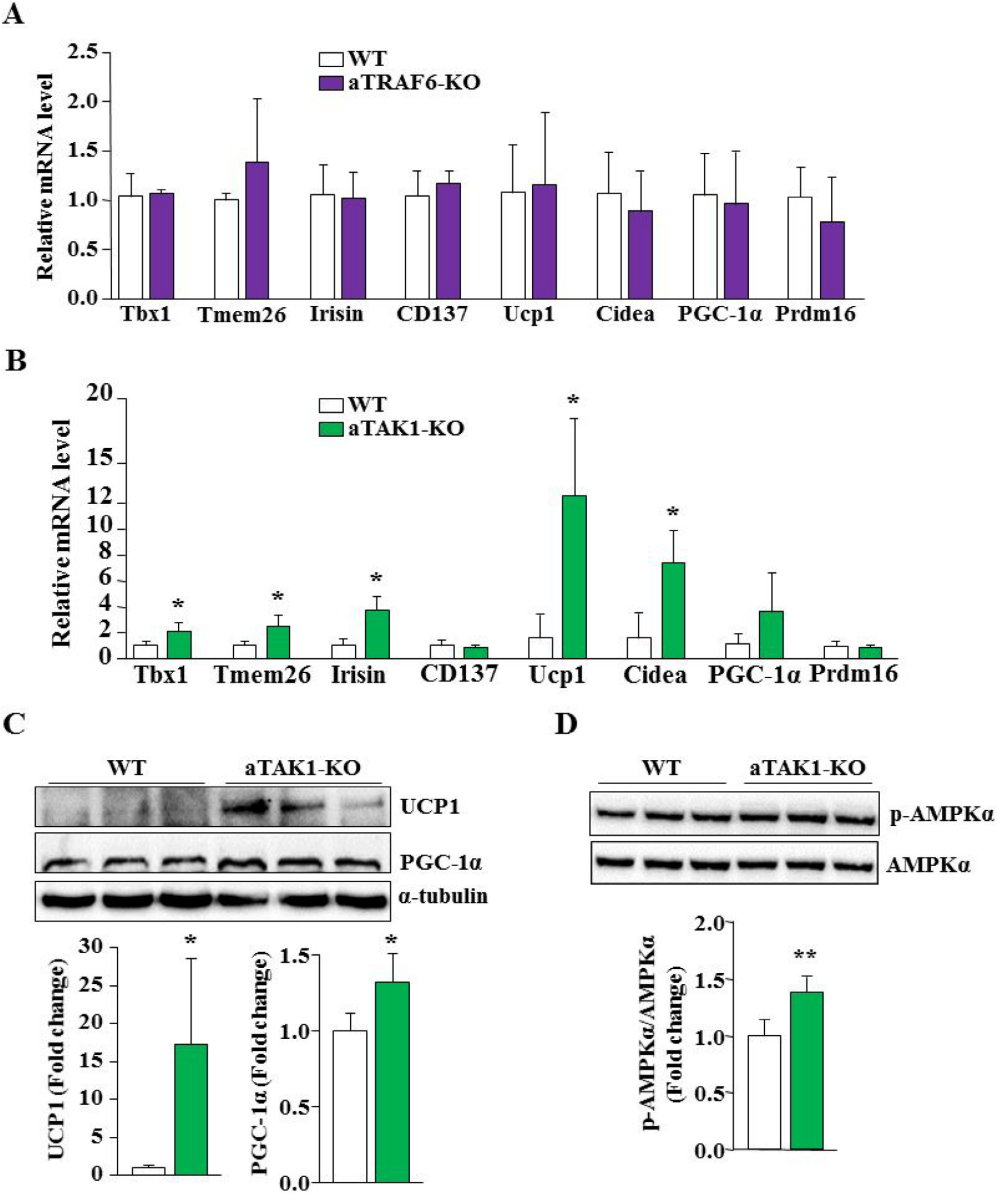
Deletion of TAK1 promotes browning of WAT in mice (**A**) eWAT was isolated from 9-week old littermate WT and aTRAF6-KO mice and the relative expression of beige cell markers and brown adipose tissue (BAT) specific genes was determined by QRT-PCR assay (*n* = 3–5 per group). (**B**) eWAT was isolated from 9-week old WT and aTAK1-KO mice and the relative expression of beige cell markers and BAT specific genes was determined by QRT-PCR assay (*n* = 3–4 per group). eWAT was isolated from 9-week old WT and aTAK1-KO mice and the levels of UCP1, PGC-1α and unrelated protein α-tubulin were measured by performing Western blot. (**C**) Representative immunoblots for UCP1 and PGC-1α and densitometry quantification of bands are presented here (*n* = 4 in each group). (**D**) Levels of phosphorylated and total AMPKα protein in eWAT from WT and aTAK1-KO mice (*n* = 4 in each group). ^*^p < 0.05, and ^**^p < 0.01, from corresponding WT mice by unpaired *t*-test.

We next investigated whether adipocyte-specific deletion of TRAF6 or TAK1 affects the transition of white fat into beige fat. We compared the mRNA levels of various markers of beige fat and BAT (e.g. Tbx1, Tmem26, Irisin, CD137, Ucp1, Cidea, PGC-1α, and Prdm16) in eWAT of aTRAF6-KO or aTAK1-KO mice with their corresponding WT mice. There was not a significant difference in any of these markers of beige and brown fats in the eWAT of WT and aTRAF6-KO mice, suggesting that deletion of TRAF6 does not affect white fat to beige fat transition in mice (Figure 4A). By contrast, we found a significant increase in mRNA levels of: Tbx1, Tmem26, Irisin, Ucp1, and Cidea in eWAT of aTAK1-KO mice compared to their littermate WT mice (Figure 4B). Although not statistically significant, we found a trend towards increased mRNA levels of PGC-1α in eWAT of aTAK1-KO mice (Figure 4B). By performing Western blot, we also measured the protein levels of UCP1, which is exclusively expressed in beige and brown fat [5, 8]. The UCP1 protein was undetectable in eWAT of WT and aTRAF6-KO mice (data not shown). Consistent with QRT-PCR results, we found a drastic increase in UCP1 protein levels in eWAT of aTAK1-KO mice (Figure 4C). In addition, the levels of the PGC-1α protein, which positively regulates browning of WAT [38, 39], were also significantly higher in eWAT of aTAK1-KO mice compared to WT mice (Figure 4C). Several studies have suggested that activation of AMPK promotes the browning of WAT [40–42]. To understand the signaling mechanisms by which inactivation of TAK1 causes transition of white to beige adipocytes, we measured the levels of phosphorylated AMPK. Interestingly, our results showed that the phosphorylation of AMPK was significantly increased in eWAT of aTAK1-KO mice compared to their corresponding controls (Figure 4D). Taken together, these results suggest that while TRAF6 has no major role, TAK1 negatively regulates the thermogenic program in WAT.

### Adipocyte-specific roles of TRAF6 and TAK1 in HFD-induced weight gain and glucose metabolism in mice

**Figure 5:**
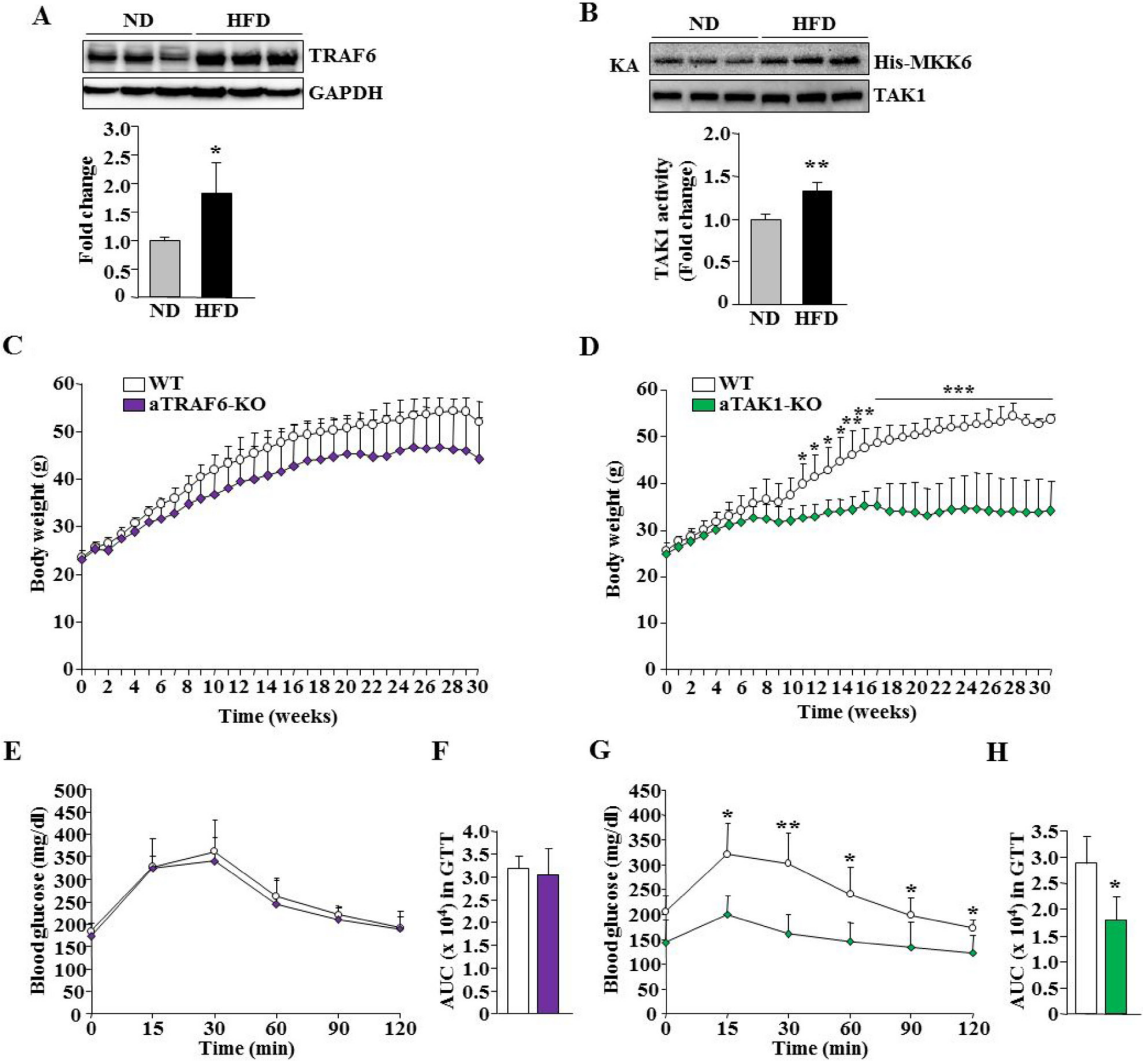
Effect of adipocyte-specific ablation of TRAF6 and TAK1 on HFD-induced obesity eWAT was isolated from mice fed with normal diet/chow (ND) and HFD and the levels of TRAF6 and unrelated protein GAPDH were measured by performing Western blot. (**A**) Representative immunoblots and densitometry analysis of bands are presented here. (**B**) Enzymatic activity of TAK1 in eWAT of ND or HFD-fed mice (*n* = 3 in each group for A and B). ^*^p < 0.05 from WT mice fed with ND by unpaired *t*-test. (**C**) Growth curve of WT and aTRAF6-KO mice fed with HFD (*n* = 4–5 per group). (**D**) Growth curve of WT and aTAK1-KO mice fed with HFD (*n* = 3–6 per group). (**E**) Blood glucose levels at indicated time points after intraperitoneal injection of glucose in glucose tolerance test (GTT) and (**F**) area under the curve (AUC) assessment for GTT (*n* = 4–5 per group) in WT and aTRAF6-KO mice fed with HFD for 30 weeks. (**G**) Blood glucose levels at indicated time points after intraperitoneal injection of glucose in GTT, and (**H**) AUC assessment for GTT in WT and aTAK1-KO mice fed with HFD for 30 weeks (*n* = 3–6 per group). ^*^p < 0.05 and ^**^p < 0.01 from WT mice by unpaired *t*-test.

While we found deletion of TAK1, but not TRAF6, reduced the survival of adipocytes and eWAT mass and improved markers of thermogenesis in naïve conditions, both TRAF6 and TAK1 are important mediators of many proinflammatory signaling pathways [13, 16, 18]. It is possible that TRAF6 and TAK1 participate in adipocyte-derived initiation and perpetuation of inflammation upon increased caloric intake. We first investigated how the levels of TRAF6 and enzymatic activity of TAK1 are affected in eWAT of mice fed with HFD. Results showed that there was a significant increase in the levels of TRAF6 in eWAT of HFD-fed mice compared to those fed with normal diet/chow (Figure 5A). Moreover, the enzymatic activity of TAK1 was significantly elevated in eWAT of HFD-mice compared to mice fed with normal diet/chow (Figure 5B). These results provide initial evidence that activation of TRAF6 and TAK1 is perturbed in WAT in response to HFD.

We next investigated the effects of adipocyte-specific deletion of TRAF6 or TAK1 in HFD-induced obesity. At the age of 8 weeks, male aTRAF6-KO and aTAK1-KO mice and their corresponding littermate WT mice were fed with HFD and their body weight was recorded every week for up to 30 weeks. While there was a trend towards reduced body weight in aTRAF6-KO mice compared to their controls as early as 6 weeks from the start of HFD, statistical significance was not achieved even after 30 weeks on HFD (Figure 5C). In contrast, body weight of aTAK1-KO mice was significantly lower compared to their control mice at 12 weeks of the start of HFD and remained lower until 30 weeks. Indeed, WT mice continued to gain weight, whereas there was no such increase in body weight in aTAK1-KO mice from 10 weeks onward on HFD (Figure 5D).

We also performed a glucose tolerance test (GTT) on mice before and after 30 weeks of feeding the HFD. There was not a significant difference in glucose clearance capacity between 8-week old littermate WT and aTRAF6-KO or littermate WT and aTAK1-KO mice (data not shown). There also was not a significant difference in the baseline plasma level of glucose and glucose clearance capacity between control and aTRAF6-KO mice in GTT (calculated based on lean tissue mass) after 30 weeks of feeding HFD (Figure 5E, 5F). In contrast, aTAK1-KO mice had reduced glucose levels and they showed improvement in glucose clearance capacity compared with their control mice in GTT (Figure 5G, 5H). We also performed insulin tolerance test (ITT) in mice fed with HFD. Intriguingly, we did not find any significant difference in insulin-stimulated glucose clearance rates between WT and aTRAF6-KO mice or WT and aTAK1-KO mice in the ITT (data not shown) implying that insulin-independent pathways may be responsible for the enhanced glucose tolerance of aTAK1-KO mice. Taken together, these results suggest that deletion of TAK1 has a more pronounced effect on the amelioration of HFD-induced obesity and a definitive role in improving glucose metabolism in adult mice.

### Effects of adipocyte-specific ablation of TRAF6 and TAK1 on fat and lean tissue mass and whole body metabolism in HFD-fed mice

**Figure 6:**
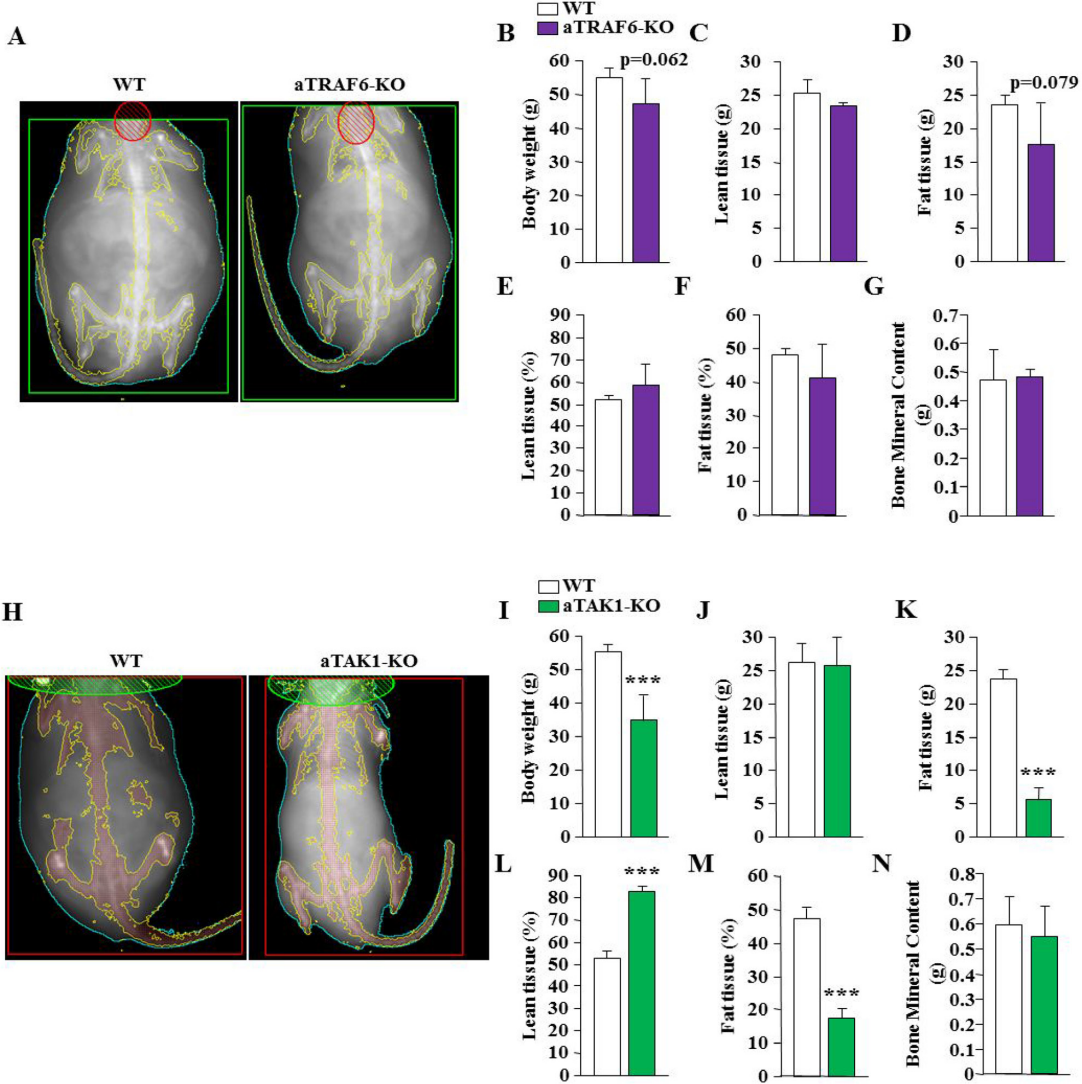
Analysis of body composition of aTRAF6-KO mice and aTAK1-KO mice fed with HFD (**A**) Representative DEXA images of WT and aTRAF6-KO mice fed with HFD for 30 weeks. (**B**) Average body weight of WT and aTRAF6-KO mice fed with HFD (*n* = 4–5 per group). Quantitative estimation of (**C**) lean tissue mass, (**D**) fat tissue mass, (**E**) percentage of body lean tissue, (**F**) percentage of body fat tissue, and (**G**) bone mineral content evaluated by whole body DEXA analysis of WT and aTRAF6-KO mice fed with HFD (*n* = 4-5 per group). (**H**) Representative DEXA images of WT and aTAK1-KO mice fed with HFD for 30 weeks. (**I**) Average body weight of WT and aTAK1-KO mice fed with HFD (*n* = 3–6 per group). Quantitative estimation of (**J**) lean tissue mass, (**K**) fat tissue mass, (**L**) percentage of body lean tissue, (**M**) percentage of body fat tissue, and (**N**) bone mineral content of WT and aTAK1-KO mice fed with HFD (*n* = 3–6 per group). ^***^p < 0.001 from corresponding WT mice by unpaired *t*-test.

To further understand the effects of adipocyte-specific deletion of TRAF6 or TAK1 on HFD-induced obesity, we measured fat and lean tissue mass by performing DEXA. While no statistical significance was achieved (*p* = 0.06), the body weight of aTRAF6-KO mice appeared to be lower than littermate WT mice after 30 weeks on HFD (Figure 6A, 6B). Despite not being statistically significant, there was a trend towards reduced fat tissue mass and increased lean tissue mass in aTRAF6-KO mice compared with WT mice after 30 weeks on HFD (Figure 6C–6F). By contrast, we found that overall body weight and fat tissue weight were significantly reduced in aTAK1-KO mice compared with their corresponding WT mice after 30 weeks of HFD (Figure 6H, 6I). Our DEXA analysis also showed a significant reduction in fat tissue mass and increase in the percentage of lean tissue mass in aTAK1-KO mice compared with WT mice (Figure 6J–6M). Adipocyte-specific deletion of TRAF6 or TAK1 had no effect on bone mineral content in mice fed with HFD (Figure 6G, 6N).

**Figure 7:**
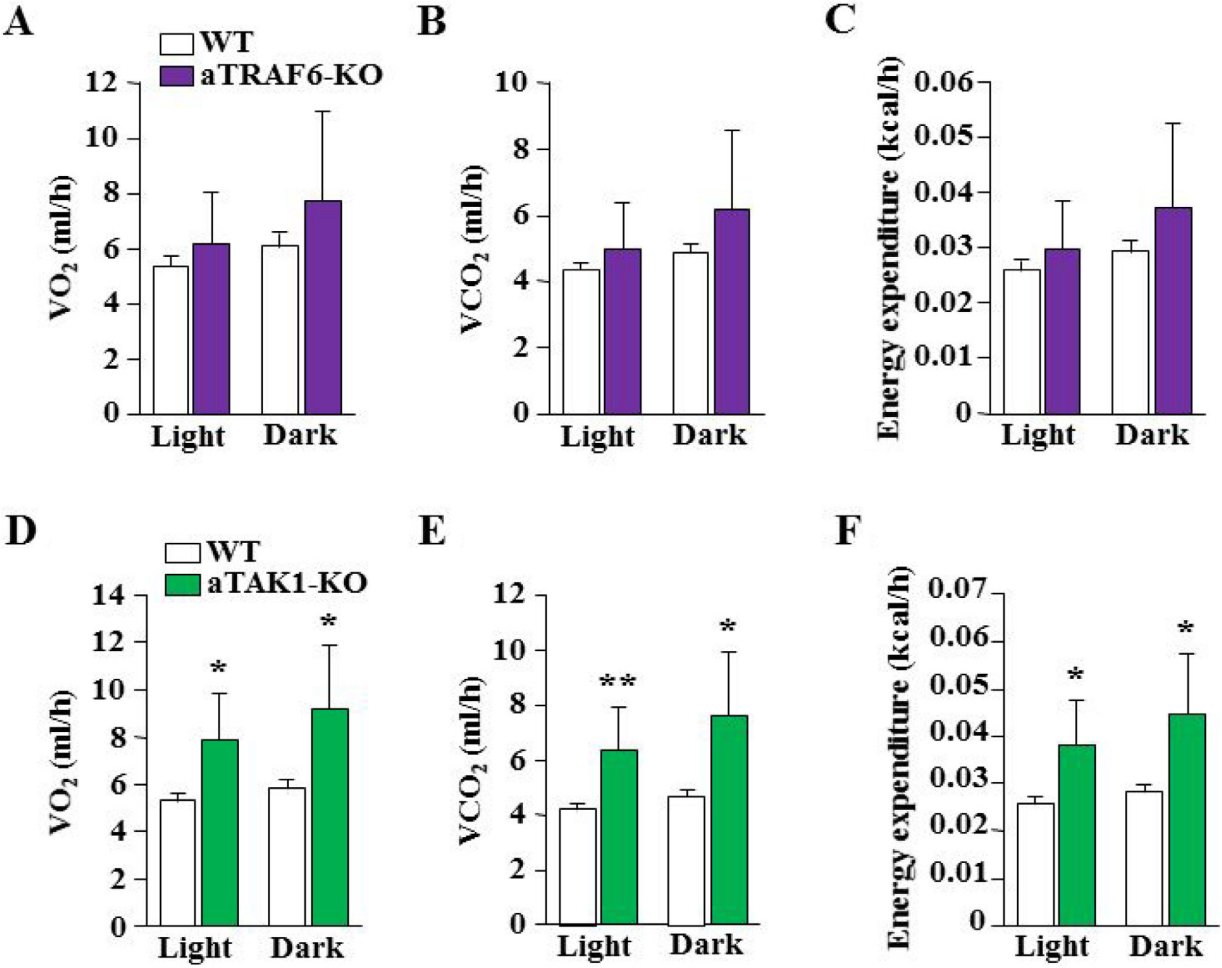
Effect of target deletion of TRAF6 or TAK1 on energy expenditure in mice in response to HFD Average light and dark phases (**A**) oxygen consumption rate (VO_2_), (**B**) carbon dioxide production rate (VCO_2_), and (**C**) whole body energy expenditure between WT and aTRAF6-KO mice fed with HFD for 30 weeks (*n* = 4–5 per group). (**D**) Average light and dark phases VO_2_, (**E**) VCO_2_, and (**F**) and whole body energy expenditure between WT and aTAK1-KO mice fed HFD for 30 weeks (*n* = 3–6 per group). ^*^p < 0.05 and ^**^p < 0.01 from corresponding WT mice by unpaired *t*-test.

To further assess the impact of adipocyte-specific deletion of TRAF6 and TAK1 in HFD-fed mice, we analyzed several aspects of behavior relevant to energy homeostasis by indirect calorimetry. We found no significant difference in food uptake between aTRAF6-KO mice or aTAK1-KO mice compared to their corresponding WT mice (data not shown). Moreover, there was no significant difference in the rate of O_2_ consumption or CO_2_ production during light and dark phases of activity between aTRAF6-KO mice and WT mice fed with HFD (Figure 7A, 7B). As a result, there was no significant difference in energy expenditure between aTRAF6-KO mice and WT mice (Figure 7C). By contrast, we found that the rate of O_2_ consumption and the rate of CO_2_ production during both light and dark phases were significantly increased in aTAK1-KO mice compared with their littermate WT mice after 30 weeks on HFD (Figure 7D, 7E). This resulted in an increased energy expenditure in aTAK1-KO mice compared to WT mice (Figure 7F).

### Effect of ablation of TRAF6 or TAK1 on adipocyte number and hypertrophy in HFD-fed mice

**Figure 8:**
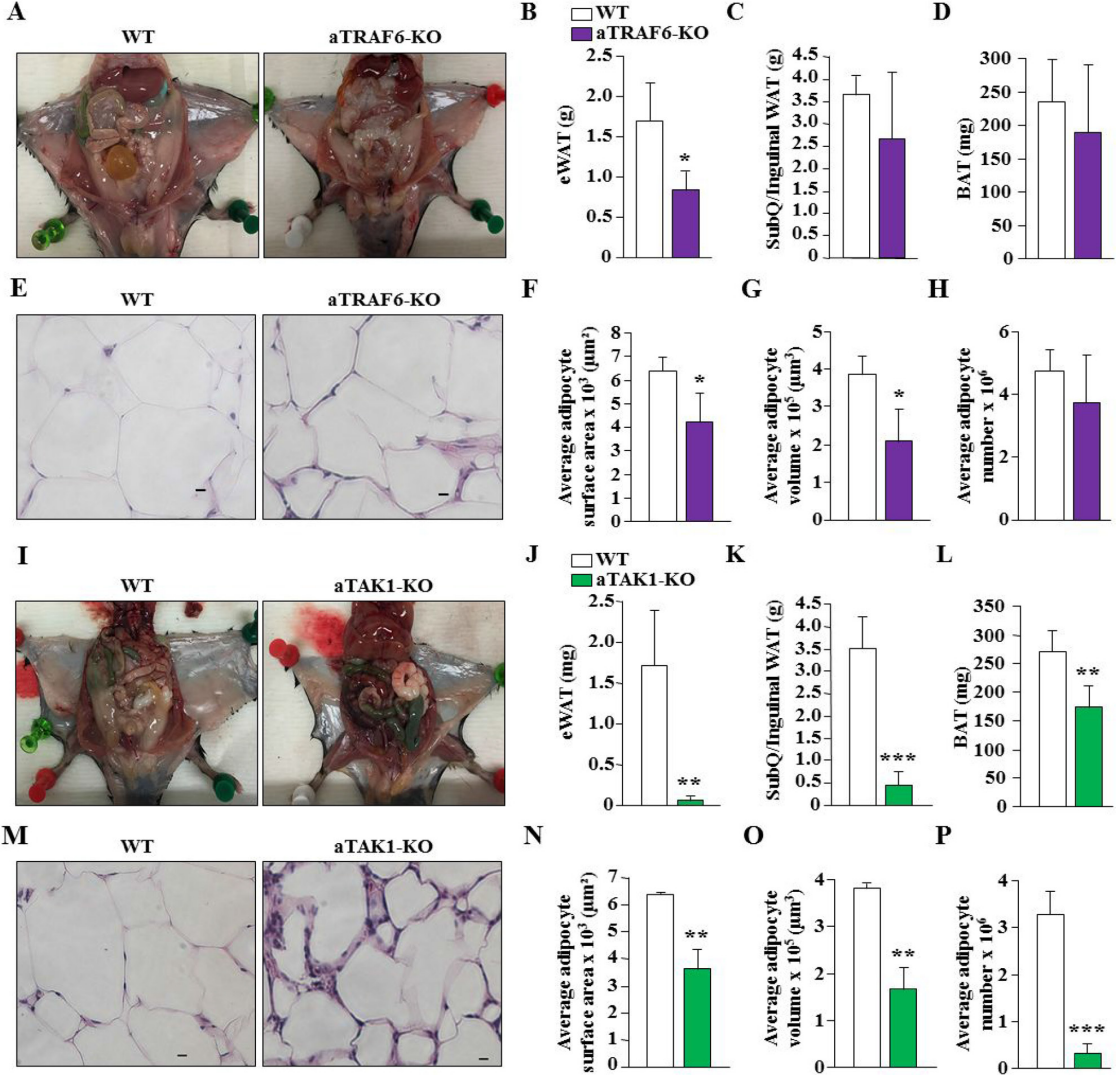
Effect of targeted ablation of TRAF6 or TAK1 on adipocyte hypertrophy and number in HFD-fed mice (**A**) Representative H&E-stained photomicrographs demonstrating epididymal and subcutaneous (SubQ)/inguinal fat in WT and aTRAF6-KO mice fed with HFD for 30 weeks. Quantitative analysis of wet weight of (**B**) epididymal WAT (eWAT), (**C**) SubQ/inguinal WAT, and (**D**) interscapular BAT from WT and aTRAF6-KO mice fed with HFD (*n* = 4–5 per group). (**E**) Representative H&E-stained images of eWAT isolated from WT and aTRAF6-KO mice fed with HFD. Scale bars: 20 μm. Quantitative analysis of (**F**) average adipocyte surface area, (**G**) average adipocyte volume, and (**H**) average adipocyte number in eWAT of WT and aTRAF6-KO mice fed with HFD (*n* = 3 per group). (**I**) Representative photomicrographs showing epididymal and subcutaneous (SubQ)/inguinal fat in WT and aTAK1-KO mice fed with HFD for 30 weeks. Quantitative analysis of weight of (**J**) eWAT, (**K**) SubQ/inguinal WAT, and (**L**) interscapular BAT from WT and aTAK1-KO mice fed with HFD (*n* = 3–6 per group). (**M**) Representative H&E-stained images of eWAT isolated from WT and aTAK1-KO mice fed HFD. Scale bars: 20 μm. (**N**) Average adipocyte surface area, (**O**) average adipocyte volume, and (**P**) average adipocyte number in eWAT of WT and aTAK1-KO mice fed with HFD (*n* = 3 per group). ^*^p < 0.05, ^**^p < 0.01, and ^***^p < 0.001 from corresponding WT mice by unpaired *t*-test.

We also investigated whether there was any difference in wet weight of individual WAT and BAT depots, size, and number of adipocytes in aTRAF6-KO mice and aTAK1-KO mice compared to their WT mice after 30 weeks on HFD. Interestingly, eWAT appeared considerably smaller in aTRAF6-KO mice compared to littermate control mice (Figure 8A). Quantitative estimation also showed that eWAT weight was significantly reduced, whereas SubQ/inguinal WAT and interscapular BAT weights were unaffected in aTRAF6-KO mice compared with littermate WT mice (Figure 8B–8D). We next performed H&E staining of eWAT of WT and aTRAF6-KO mice (Figure 8E) and quantified adipocyte surface area, volume, and number of adipocytes. There was a significant decrease in the surface area and volume of adipocytes in eWAT of aTRAF6-KO mice compared with WT mice (Figure 8F, 8G). However, the number of adipocytes remained comparable between eWAT of WT and aTRAF6-KO mice after 30 weeks on HFD (Figure 8H). In comparison to aTRAF6-KO mice, we found that wet weights of epididymal and SubQ/inguinal WAT as well as interscapular BAT were significantly reduced in aTAK1-KO mice compared with their corresponding WT mice fed with HFD (Figure 8I–8L). H&E staining and morphometric analysis showed that adipocyte surface area and volume and the number of adipocytes were significantly reduced in eWAT of aTAK1-KO mice compared with WT mice (Figure 8M–8P). Collectively, these results suggest that while deletion of TRAF6 has a lessened impact in reducing obesity, TAK1 drastically reduces HFD-induced increase in adipose tissue mass, potentially through reducing adipocyte hypertrophy and survival.

## DISCUSSION

Adipose tissue was initially considered a passive repository for triglyceride accumulation in adipocytes. However, it is now established that adipose tissue is a metabolically dynamic organ that produces a variety of secretory factors, such as: adipsin, leptin, resistin, TNFα, IL-6, adiponectin, fatty acids and prostaglandins; these factors exert multiple effects at both the local and the systemic levels [5]. With the exception of adiponectin, the expression and secretion of many of these factors become elevated during obesity, resulting in a state of chronic low-grade inflammation and metabolic abnormalities [43]. Accumulating evidence also suggests that adipose tissue undergoes continuous self-renewal through the differentiation of pre-adipocytes from committed stem cells residing in adipose tissue and also from transdifferentation of several other cell types into adipocytes [5, 8, 44]. While adipocyte number remains relatively constant in humans during adulthood, about 10% of fat cells are renewed annually in lean individuals and during the early stages of obesity [45]. However, significantly higher number of adipocytes has been found in the adipose tissue of severely obese individuals [45]. Reducing the number of adipocytes and diminishing the inflammatory response can be an important strategy to treat obesity and metabolic syndromes.

TRAF6 and TAK1 are two important proximal signaling molecules that regulate the survival and fate of a number of cell types [18, 29, 36]. TRAF6 interacts with the cytoplasmic domains of TLRs, CD40, T-Cell receptors (TCR), IL-1R, IL-17R, and TGF-β1 receptors in both immune and non-immune cells. TRAF6 also possesses non-conventional E3 ubiquitin ligase activity through which it facilitates a wide array of protein-protein interactions, resulting in the context-dependent activation of MAPKs, phosphoinositide 3-kinase, and interferon regulatory factor pathways [18]. Upon activation, TRAF6 attaches K63-linked ubiquitin chains to lysine residues on various target proteins, including itself. These ubiquitin chains enable the formation of complexes that lead to kinase activation through adapters, such as TAB2/3, which contain ubiquitin-binding domains. TAB2/3 recruit the kinase TAK1, which phosphorylates downstream kinases, such as IKKβ and MKKs, eventually leading to the activation of the proinflammatory transcription factors NF-κB and AP-1, respectively [29, 36]. Indeed, it has been consistently found that the activation of TAK1 through TRAF6 is a critical step for the activation of several downstream signaling pathways in response to many cytokines and microbial products [18]. Other receptors, including the TNF receptors, recruit other members of the TRAF family, such as TRAF2 and TRAF3, to activate the TAK1 signalosome [46].

In this study, we investigated the role of TRAF6 and TAK1 in adipocyte formation and HFD-induced obesity. Our results demonstrate that the ablation of TRAF6 in adipocytes from birth did not have any effect on the WAT or BAT mass or adipocyte number, suggesting that TRAF6 is dispensable for the formation of adipose tissues. By contrast, we found that WAT mass especially eWAT was significantly reduced in aTAK1-KO mice (Figure 1). Similar to these findings, Sassmann-Schweda et al have recently reported that tamoxifen-inducible inactivation of TAK1 causes adipocyte cell death through apoptosis [30]. We observed that there was a significant decrease in the number of adipocytes in eWAT of aTAK1-KO mice in the naïve state. Moreover, the levels of cleaved caspase-3, which acts as both an initiator and an executor of the apoptotic process, were elevated in eWAT of aTAK1-KO mice, further suggesting that TAK1 prevents apoptotic adipocyte death (Figure 2). Even though our analysis and recently published report [30] suggest that apoptosis is a prominent mechanism, there is also a possibility that deletion of TAK1 affects adipogenesis and transdifferentiation potential of progenitor cells leading to reduced amounts of WAT in a TAK1-KO mice.

Both TRAF6 and TAK1 play an important role in the activation of inflammatory pathways such as MAPK and NF-κB [18]. Intriguingly, we found that there was a significant increase in the transcript levels of various inflammatory cytokines and/or their receptors and markers of macrophages in eWAT of aTAK1-KO mice, but not in aTRAF6-KO mice (Figure 3). Although the exact reasons remain unknown, it is possible that continued cell death in the adipose tissues of aTAK1-KO mice initiates an inflammatory response to remove the dead adipocytes. There are several other reports suggesting that deletion of TAK1 causes a severe inflammatory response and oxidative stress in other tissues such as liver and skin [47–50]. However, there is also the possibility that deletion of TAK1 perturbs the activation of specific signaling molecules in adipocytes, which leads to the increased expression of inflammatory molecules. TAK1 is an important signaling intermediate that causes the activation of JNK1/2 in mammalian cells [18]. Our results demonstrate that deletion of TAK1 significantly reduced the phosphorylation of JNK in eWAT of aTAK1-KO mice. By contrast, a small but significant upregulation in p38 MAPK was noticeable (Figure 3E, 3F), suggesting that deletion of TAK1 perturbs the activation of MAPKs in adipocytes.

NF-κB is a major transcription factor that induces the expression of many inflammatory molecules as well as cell survival molecules [28]. Our results demonstrate that the levels of phosphorylated IkBα and p65 proteins, the markers of activation for the canonical NF-κB pathway [46], were reduced in eWAT of aTAK1-KO mice. Intriguingly, we observed that levels of p52 protein were significantly elevated in eWAT of aTAK1-KO mice (Figure 3E, 3F). While TAK1 does not directly regulate the non-canonical NF-κB pathway [46], it is likely that the inhibition of canonical NF-κB signaling leads to the activation of non-canonical NF-κB pathway as a part of compensatory mechanism in eWAT of aTAK1-KO mice. Since canonical NF-κB signaling regulates the expression of pro-survival molecules [28], inhibition of this arm of NF-κB may be an important mechanism for the increased cell death observed in eWAT of aTAK1-KO mice.

An important emerging approach to reduce obesity is to activate the thermogenic program in tissues such as the skeletal muscle and beige/BAT [3]. While there is a preponderance of data supporting the existence of brown adipocytes within the WAT of both rodents and humans [5, 8, 51], signaling pathways that regulate the appearance of these brown adipocytes remain largely obscure. Interestingly, we found that deletion of TAK1, but not TRAF6, improves the amount of interscapular BAT in mice in naïve conditions (Figure 1). More importantly, we found that the markers of beige/brown fat were significantly up-regulated in eWAT of aTAK1-KO mice suggesting that deletion of TAK1 improves browning of WAT (Figure 4). A recent study demonstrated that inhibition of Notch signaling improves the browning of WAT [6, 52]. TAK1 regulates the Notch pathway through signaling cross-talk [53]. However, our analysis showed that there was not a difference in the levels of Notch 1 intracellular domain (N1ICD) or the mRNA levels of Notch target genes such as: Hes1, Hes6, Hey1 and HeyL in eWAT of WT and aTAK1-KO mice (data not shown).

AMPK is a master regulator of cellular energy homeostasis that directly phosphorylates metabolic enzymes and nutrient transporters and promotes the transactivation of nuclear genes involved in mitochondrial biogenesis and function through PGC-1α [54]. Indeed, the activation of AMPK promotes the browning of WAT in response to specific stimuli [40–42]. There are also reports suggesting that p38 MAPK is essential for phosphorylating transcription factors that drive PGC-1α and UCP1 expression and browning of white adipose tissue in response to cyclic AMP (cAMP) or cardiac natriuretic peptides [55–57]. Interestingly, we found that there are increased levels of phosphorylated AMPK and p38 MAPK along with increased abundance of PGC-1α in eWAT of aTAK1-KO mice (Figures 3E, 3F, 4C, 4D). Recently, the thermogenic beige fat circuitry was identified to contain alternatively activated M2 macrophages, which promotes browning of white fat through production of catecholamines [58]. We observed that the markers of M2 macrophages were significantly increased in eWAT of aTAK1-KO mice. IL-10 is a critical cytokine, which promotes the M2 macrophage phenotype [59]. Consistent with increased expression of M2 macrophage markers, we also found a significant increase in the mRNA levels of IL-10 in eWAT of aTAK1-KO mice (Figure 3D). By contrast, deletion of TRAF6 had no effect on the markers of M2 macrophages or IL-10 expression in WAT (Figure 3B). Although the exact mechanisms by which deletion of TAK1 increases phosphorylation of AMPK; increases levels of PGC-1α; and improves the abundance of M2 macrophages, remain unknown, they may collectively promote browning of eWAT in aTAK1-KO mice.

We also compared the role of TRAF6 and TAK1 in HFD-induced obesity and glucose metabolism. A previous study has shown that the inhibition of CD40-TRAF6 signaling in MHCII^+^ cells reduced adipose tissue inflammation and hepatosteatosis and improved insulin sensitivity in mice fed with HFD [60]. Our results suggest that while aTRAF6-KO mice gained comparatively less weight, the effect was not significant (Figure 5C). Moreover, there was not a difference in the glucose clearance capacity or energy expenditure between WT and aTRAF6-KO mice (Figures 5E, 5F, 7C). However, we observed a significant reduction in wet weight and adipocyte hypertrophy in eWAT of aTRAF6-KO mice (Figure 8), suggesting that the inhibition of TRAF6 in adipocytes has a relatively lessened impact on HFD-induced weight gain. Since inflammation contributes significantly to HFD-induced obesity, the inhibition of TRAF6 signaling in inflammatory immune cells may be more effective in prevention of HFD-induced obesity as previously reported [60].

Intriguingly, while there was a significant increase in the amounts of interscapular BAT and a significant decrease in eWAT in naïve conditions (Figure 1), we found that the amounts of WAT as well as BAT were significantly reduced in HFD-fed aTAK1-KO mice compared with WT mice (Figure 8). Although deletion of TAK1 increases the levels of beige markers in eWAT of aTAK1-KO, it is not clear whether TAK1 has a role in BAT adipogenesis. Future studies should determine the role of TAK1 in development of BAT. However, the reduced amounts of WAT and BAT in HFD-fed aTAK1-KO mice could be attributed to increased adipocyte mortality. TAK1 is known to promote cell survival through the activation of NF-κB pathway [29, 36]. Indeed, whole body TAK1-KO or p65-KO mice are embryonically lethal due to excessive TNFα-induced apoptosis in liver [29, 36, 61]. It is possible that in naïve conditions, the levels of inflammatory cytokines are not high enough to cause apoptosis in interscapular BAT. HFD causes inflammatory response and increases the levels of various inflammatory cytokines including TNFα in both adipose tissues and circulation [14, 15], which can stimulate adipocyte death not only in WAT, but also in BAT of aTAK1-KO mice. Consistent to this presumption, we found negligible amounts of eWAT in HFD-fed aTAK1-KO mice along with a significant decrease in the amounts of BAT (Figure 8I-8L) and a reduced number of adipocytes in eWAT of aTAK1-KO mice (Figure 8P). Since deletion of TAK1 also improves browning of eWAT, protection of aTAK1-KO mice from HFD-induced obesity could also be attributed to increased expenditure of energy in adipose tissues. Indeed, our analysis showed a higher rate of O_2_ consumption and CO_2_ production in aTAK1-KO mice (Figure 7D-7F).

In summary, our results demonstrate that even though both TRAF6 and TAK1 are common components of several signaling pathways, inhibition of TAK1, but not TRAF6, reduces adipocyte survival and improves browning of WAT in mice. Future studies will identify the role of these signaling molecules in other cell types found in the vascular stromal fraction of adipose tissues.

## MATERIALS AND METHODS

### Animals

Adipocyte-specific TAK1 knockout mice (that is, aTAK1-KO) and adipocyte-specific TRAF6 knockout mice (that is, aTRAF6-KO) were generated by crossing adiponectin (Adipoq)-Cre mice (Jax Strain: B6;FVB-Tg(Adipoq-Cre)1Evdr/J) with floxed TAK1 and floxed TRAF6 mice, respectively. All mice were in the C57BL6 background and their genotype was determined by PCR from tail DNA. Mice were housed in a 12h light/12h dark cycle and given water and food ad libitum. For one experiment, 8-week old male mice were placed on normal diet/chow (ND) or a 60% HFD (Research Diets, cat# D12492). Body weights were recorded weekly. All experimental protocols with mice were approved in advance by the Institutional Animal Care and Use Committee (IACUC) and Institutional Biosafety Committee at the University of Louisville (IACUC numbers: 13097 and 16663).

### Immunohistochemistry and morphometric analysis

For histological analysis of epididymal WAT (eWAT), the tissue was fixed in 10% neutral formalin for 24 h prior to embedding in paraffin. For the assessment of tissue morphology, 5 μm (paraffin-embedded) thick transverse sections of tissue were stained with hematoxylin and eosin (H&E), and the photomicrographs were captured with a microscope (Eclipse TE 2000-U; Nikon, Melville, NY, USA) fitted with a digital camera (Digital Sight DS-Fi1; Nikon) and NIS Elements BR 3.00 software (Nikon). The images were quantified using Fiji software (U.S. National Institutes of Health, Bethesda, MD, USA) to determine surface (S) area of adipocytes in eWAT. Adipocyte volume (V) was calculated from H&E-stained paraffin sections using the formula V = 4 S^3/2^/(3 √ π) where S refers to the previously calculated adipocyte surface area. Finally, the number of adipocytes was determined by calculating the ratio of eWAT volume (obtained from multiplying eWAT weight and adipose tissue density factor 0.92 g/cm^3^) to adipocyte volume (V) as described [62].

### Glucose tolerance test (GTT)

The GTT was performed following a method as previously described [63]. In brief, mice were fasted for 6h before providing an intraperitoneal injection of sterile glucose (1g/kg lean body mass in saline). The blood glucose levels were monitored at various time points for up to 2h. Blood samples were collected from the tail veins, and blood glucose was measured with a handheld glucometer device (Accu-Chek Aviva Plus; Roche Diagnostics, Indianapolis, IN, USA). The area under the curve was calculated according to the trapezoidal rule as previously described [63]. In brief, the area under the curve was divided between 2 designated values on the x axis into small segments, and each area was calculated from its respective geometrical formula. Finally, the area of the individual segment was added to obtain the total area under the curve.

### Metabolic cage analysis

Using a physiological/metabolic cage system (TSE LabMaster, Chesterfield, MO, USA), the volume of O_2_ consumed (VO_2_) and volume of carbon dioxide produced (VCO_2_), as well as the whole-body energy expenditure and food and water intake were measured. After the system was calibrated against standard gas mixtures, individual mice were transferred to an experimental cage containing specialized lids for water and food delivery and inlet/outlet airflow. The measurement was performed for 24h at room temperature. Data collected during the light and dark phases were used for statistical analysis.

### Analysis of body composition

The fat and lean body mass composition, as well as bone mineral content of mice were determined by dual-energy X-ray absorptiometry (DEXA) using a mouse densitometer (DEXA; PIXImus2; Lunar, Madison, WI, USA) after HFD feeding.

### Western blot

For measuring protein abundance, eWAT homogenates were prepared in lysis buffer [50 mM Tris pH 7.5, 500 mM NaCl, 1% IGEPAL, 20% glycerol, 2 mM EDTA, 1 mM dithiothreitol, 1 mM sodium orthovanadate and protease inhibitors]. The samples were centrifuged at 14,000 × g for 20 min at 4°C, and the supernatants were used for subsequent analyses. Seventy (70) μg of protein was resolved on 10% SDS-polyacrylamide gel, then blotted onto 0.45 mm nitrocellulose membrane and probed overnight using specific primary antibody.

The following antibodies were used for Western blotting: anti-TRAF6 (MBL International; cat# 597), anti-TAK1 (Cell Signaling Technology; cat# 4505), anti-Cleaved Caspase-3 (Cell Signaling Technology; cat# 9664), anti-phospho-JNK1/2 (Cell Signaling Technology, cat# 9251), anti-JNK1/2 (Cell Signaling Technology, cat# 9252), anti-phospho-p38 MAPK (Cell Signaling Technology, cat# 9211), anti-p38 MAPK (Cell Signaling Technology, cat# 9212), anti-phospho-IκBα (Cell Signaling Technology, cat# 2859), anti-IκBα (Cell Signaling Technology, cat# 4812), anti-phospho-p65 (Cell Signaling Technology, cat# 3033), anti-p65 (Cell Signaling Technology, cat# 8242), anti-p100/p52 (Cell Signaling Technology, cat# 4882), anti-UCP1 (Santa Cruz Biotechnology; cat# sc-293418), anti-PGC-1α (Santa Cruz Biotechnology; cat# sc-13067), anti-phospho-AMPKα (Cell Signaling Technology, cat# 2535), anti-AMPKα (Cell Signaling Technology, cat# 2532), anti-GAPDH (Cell Signaling Technology; cat# 2118), and anti-α-tubulin (Cell Signaling Technology; cat# 2125). Bound antibodies were detected by secondary antibodies conjugated to horseradish peroxidase (Cell Signaling Technology). Signal detection was performed by an enhanced chemiluminescence detection reagent (Bio-Rad). Approximate molecular masses were determined by comparison with the migration of prestained protein standards (Bio-Rad). Band intensities were quantified using Fiji software (NIH).

### Immunoprecipitation and *in vitro* kinase assay

eWAT were isolated from normal and HFD-fed mice and eWAT homogenates were prepared in lysis buffer [50 mM Tris pH 7.5, 500 mM NaCl, 1% IGEPAL, 20% glycerol, 2 mM EDTA, 1 mM dithiothreitol, 1 mM sodium orthovanadate and protease inhibitors] and protein concentration was measured. Five hundred to 600 μg of protein was immunoprecipitated with 1 μg TAK1 (D94D7) rabbit monoclonal antibody (Cell Signaling Technology; cat #5206) and the immune complex was collected using protein A-Sepharose beads. After washing two times with lysis buffer and two times with kinase buffer (50 mM HEPES (pH 7.4), 10 mM MgCl2, and 1 mM DTT), the beads were suspended in 20 μl of kinase assay mixture containing 50 mM HEPES (pH 7.4), 20 mM MgCl2, 2 mM DTT, 5 μCi of [γ-32P]ATP, 1 μM unlabeled ATP, and 2 μg of His-MKK6 as substrate. After incubation at 37°C for 15 min, the reaction was terminated by boiling with 20 μl of 2× Laemmli sample buffer for 3 min. Finally, the protein was resolved on a 10% polyacrylamide gel, the gel was dried, and the radioactive bands were visualized by exposing to a PhosphorImager screen and quantified using Fiji software (NIH).

### qRT-PCR assay

For quantitative RT-PCR, total RNA was extracted from frozen tissues using QIAzol lysis reagent (Qiagen), and purified with the RNeasy lipid tissue kit (Qiagen). Any contaminating DNA was removed using the DNA-free kit from Ambion (Austin, TX). The quantity of RNA was analyzed using NanoDrop instrumentation (NanoDrop Technologies, Wilmington, DE). Purified RNA (4 μg) was used to synthesize first strand of the cDNA by reverse transcription system using Ambion's oligo-dT primer and Qiagen's Omniscript reverse transcriptase according to the manufacturer's instructions. The first strand of cDNA reaction (1 μl from 40 μl cDNA stock) was subjected to real-time PCR amplification using gene-specific primers. The primers were designed using Vector NTI Xi software (Invitrogen) and their sequence is available on request. Quantification of mRNA was done with the SYBR Green method using ABI Prism 7300 Sequence Detection System (Applied Biosystems, Foster City, CA). Approximately, 25 μl of reaction volume was used for the real-time PCR assay that consisted of 2 × (12.5 μl) Brilliant SYBR Green QPCR Master Mix (Applied Biosystems), 400 nM of primers (1 μl each from the stock), 11 μl of water and 0.5 μl of template. The thermal conditions consisted of an initial denaturation at 95°C for 10 min followed by 40 cycles of denaturation at 95°C for 15 s, annealing and extension at 60°C for 1 minute and a final step melting curve of 95°C for 15 s, 60°C for 15 s and 95°C for 15 s. All reactions were carried out in duplicate to reduce variation. The data were analyzed using SDS software version 2.0, and the results were exported to Microsoft Excel for further analysis. Data normalization was accomplished using the endogenous control β-actin and the normalized values were subjected to a 2^−ΔΔCt^ formula to calculate the fold change between the control and experimental groups.

### Statistical analysis

Comparisons between 2 groups were performed with unpaired Student's *t*-test (2-tailed) and results are expressed as mean ± s.d. A value of *P* < 0.05 was considered statistically significant.

## Abbreviations

AMPK: adenosine monophosphate-activated protein kinase
AP-1: activator protein 1
BAT: brown adipose tissue
cAMP: cyclic adenosine monophosphate
Cidea: cell death-inducing DFFA-like effector a
DEXA: dual-energy X-ray absorptiometry
eWAT: epididymal white adipose tissue
GA: gastrocnemius
GAPDH: glyceraldehyde-3-phosphate dehydrogenase
GTT: glucose tolerance test
HFD: high-fat diet
H&E: hematoxylin and eosin
IκB: inhibitor of kappa B
IKK: inhibitor of kappa B (IκB) kinase
IL: interleukin
JNK: c-Jun N-terminal kinase
KO: knockout
MAPK: mitogen-activated protein kinase
MHC: major histocompatibility complex
MKK: mitogen-activated protein kinase kinase
ND: normal diet
NF-κB: nuclear factor-kappa B
N1ICD: notch 1 intracellular domain
PGC-1α: peroxisome proliferator-activated receptor coactivator-1alpha
Prdm16: PR/SET domain 16
QRT-PCR: quantitative reverse transcription polymerase chain reaction
SubQ: subcutaneous
TA: tibialis anterior
TAB: transforming growth factor-activated kinase 1-binding protein
TAK1: transforming growth factor-activated kinase 1
Tbx1: T-box 1
TCR: T-cell receptor
TGFβ1: transforming growth factor beta 1
TLRs: toll-like receptors
Tmem26: transmembrane protein 26
TNF: tumor necrosis factor
TNFR: tumor necrosis factor receptor
TRAF6: TNF receptor-associated factor 6
T2D: type 2 diabetes
UCP1: uncoupling protein-1
WAT: white adipose tissue
WT: wild-type
